# Effects of Curing Conditions on the MECHANICAL and Microstructural Properties of Ultra-High-Performance Concrete (UHPC) Incorporating Iron Tailing Powder

**DOI:** 10.3390/ma14010215

**Published:** 2021-01-04

**Authors:** Dong Lu, Jing Zhong, Baobao Yan, Jing Gong, Ziye He, Guanhua Zhang, Chengzhe Song

**Affiliations:** 1 College of Urban Construction, Wuchang University of Technology, Wuhan 430223, China; dongluhit@163.com; 2 School of Civil Engineering, Harbin Institute of Technology, Harbin 150090, China; 3 School of Materials Science and Engineering, Chang’an University, Xi’an 710064, China; 4 School of Civil Engineering and Architecture, Wuhan Polytechnic University, Wuhan 430023, China; 5 College of Architecture and Technology, Hubei Polytechnic Institute, Xiaogan 432000, China; babydog25@163.com; 6 Liaoning Provincial Transportation Planning and Design Institute Co., Ltd., Shenyang 110111, China; lnzgh123@163.com (G.Z.); songzhe0323@163.com (C.S.)

**Keywords:** ultra-high-performance concrete, iron tailing powder, early-age strength, microstructure, warm-water curing, steam curing

## Abstract

It has been reported that iron tailing powder (ITP) has the potential to partially replace cement to prepare ultra-high-performance concrete (UHPC). However, the reactivity of ITP particles in concrete largely depends on the curing method. This study investigates the effects of curing conditions on the mechanical and microstructural properties of UHPC containing ITP. To achieve this objective, three research tasks are conducted, including (1) preparing seven concrete formulations by introducing ITP; (2) characterizing their mechanical performance under different curing regimes; and (3) analyzing their microstructure by XRD patterns, FTIR analysis, and SEM observation. The experimental results show that there is an optimum ITP dosage (15%) for their application. The concrete with 15% ITP under standard curing obtains 94.3 MPa at 7 days, their early-age strength could be even further increased by ~30% (warm-water curing) and ~35% (steamed curing). The steam curing regime stimulates the activity of ITP and refines the microstructure. This study demonstrates the potential of replacing Portland cement with ITP in UHPC production.

## 1. Introduction

Ultra-high-performance concrete (UHPC) has been extensively used in construction and building engineering due to its extraordinary compressive strength of over 120 MPa and excellent durability [1]. The super performance derives from low water to binder ratio (w/b < 0.30) [2], a high quantity of cementitious materials (1100–1300 kg/m^3^), and a suitable amount of superplasticizer [3]. However, a considerable amount of CO_2_ is emitted in concrete production due to cement consumption [4], which greatly contributes to the greenhouse effect [5]. Thus, it is urgent to reduce cement consumption in concrete production with the increasing demand for environmental harmony [6].

To address this urgent issue, several researchers have attempted to replace Portland cement (PC) with supplementary cementitious materials (SCMs) in UHPC production [7]. Traditional SCMs have a pozzolanic potential [8], micro-filler effect [9], and nucleation effect [10], contribute to the strength formation, while the ordinary early-age strength, energy consumption, and high-cost [3,11], hindering their applications in some fields [3]. Therefore, low energy consumption, environment friendly, and low-cost UHPC will be highly desired in future generations in civil engineering.

It is reported that there are more than 600 million tons of iron tailings produced in China each year, and their accumulated quantity has reached ~10 billion tons [12]. Which seriously pollutes the environment and occupies land space due to the low utilization rate (~7%) in China [13]. Therefore, it is urgent to dispose of these iron tailings. The major components of the iron tailings are Fe_2_O_3_, SiO_2,_ and Al_2_O_3_ [13], and their chemical–mineralogical constitution is readily considered as an alternative material for improving the performance of concrete [14,15]. Moreover, their low-cost is conducive to application in concrete production [16,17]. Iron tailing powder (ITP), as a product of grinding iron tailings, has pozzolanic potential and accelerates the hydration of cement [17]. Furthermore, the unreacted particles can form a good grade, contributing to form the dense structure and to improving the mechanical strength of concrete [18]. Therefore, it is an ideal alternative to cementitious materials for concrete production.

To date, several relevant and representative investigations have been reported that have added ITP in cement-based materials [19]. For instance, Defáveri et al. [20] studied the mechanical properties of ITP-based composites and found that the specimen obtained high compressive (~100 MPa) and flexural strength (~20 MPa). Han et al. [12] found that the addition of ITP significantly promoted the hydration of the binder at a low w/b (0.30). Cai et al. [10] investigated the effect of fineness of ITP on the mechanical and hydration characteristics of concrete, and the test results indicated that the finer ITP could effectively enhance their strength. Most recently, an investigation carried out by Han et al. [16] reported that the specimen incorporating 30% ITP obtained ~70 MPa at 365 days. It could be said that using ITP as an alternative cementitious material in concrete related applications remains both technically and economically attractive. Although these newly developed ITP modified concretes have shown limited success, the combination effects of variable dosages of ITP with silica fume (SF) on mechanical and microstructural properties of UHPC—especially the UHPC under different curing regimes—have not been fully investigated.

The objective of this study is to gain insights into the mechanical and microstructural properties of UHPC under different curing conditions. Seven UHPC formulations are designed by different replacement levels of ITP (0–30%) and constant dosage of SF (20%), and the effects of curing conditions (standard curing, warm-water curing at 45 °C, and steam curing at 90 °C) on mechanical and microstructural properties of concrete are investigated. It is greatly significant to promote the application of ITP in UHPC preparation.

## 2. Experimental Details

### 2.1. Materials

**Figure 1:**
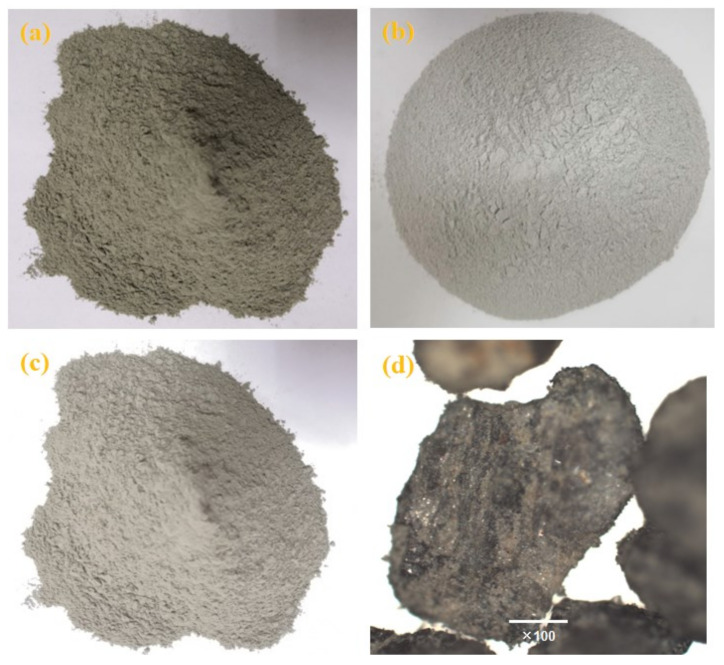
The physical appearance of (**a**) cement, (**b**) silica fume (SF), and (**c**) iron tailing powder (ITP), and (**d**) morphology of the ITP.

**Figure 2:**
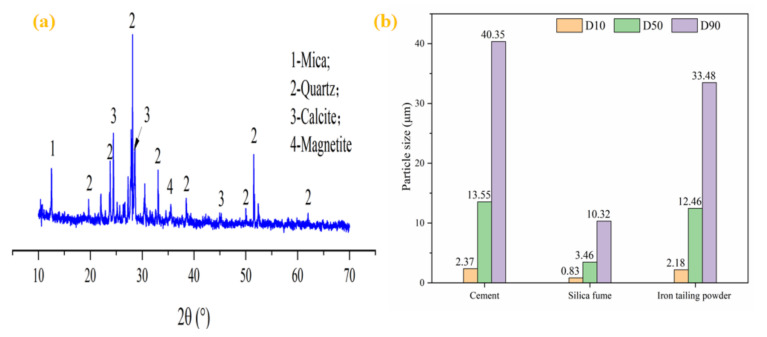
The XRD patterns of ITP (**a**) and (**b**) particle size distribution of the cementitious materials.

Portland cement (P·O 42.5), silica fume (SF), and iron tailing powder (ITP) was used in this study, as presented in Figure 1a–c. The physical properties of the cement (Table 1) were conforming to the Chinese National Standards GB 175-2007 [21]. The SF had moisture of 0.58%, a density of 2.21 g/cm^3^, and loss on ignition of 2.86%. The ITP was obtained from iron tailings after grinding for 45 min [22], it had a specific surface area of 580 m^2^/kg, a cumulative volume under 10 μm of 58.32%, and a density of 2.72 g/cm^3^ respectively. The morphology of the ITP was presented in Figure 1d shows that there were plenty of fine particles with irregular shapes. The XRD patterns of the ITP was provided in Figure 2a shows that it had an abundant calcium phase. Figure 2b shows the particle size distribution of the cementitious materials, it is clear that ITP had the lowest medium particle size (D50), which indicates that ITP had the finest particle size among raw materials. This means that the concrete containing ITP had particle dense packing, which helps the mechanical properties development of concrete [23]. Table 2 presents the chemical composition of cementitious materials by X-Ray Fluorescence (XRF9, Beijing Hongchangxin Technology Co., Ltd., Beijing, China).

Fine quartz sand with the maximum particle diameter of 0.80 mm and an apparent density of 2.542 g/cm^3^. Polycarboxylic superplasticizer with water-reducing of 35% and a solids content of 30% was added to adjust the fluidity of concrete [24].

### 2.2. Mix Design and Specimen Preparation

To investigate the combined effects of variable dosages of ITP with silica fume (SF) on mechanical and microstructural properties of concrete, seven concrete formulations were prepared. The fiber admixture was omitted, the focus of this investigation was based on mineral constituent, FTIR spectrum, and morphology analysis. Cement mortar mixtures were prepared to simulate the UHPC matrix, as recommended by Mo et al. [3]. The reference sample is labeled as C100ITP0 (Table 3). The Portland cement was substituted by ITP (Table 3) to prepare UHPC mortar (Table 4).

### 2.3. Curing Regimes

To investigate the effects of curing conditions on the mechanical and microstructural properties of concrete containing ITP, the standard curing ((20 ± 1) °C, RH ≥ 95%) according to the Chinese National Standard GB/T 17671-1999 [25] warm-water curing (45 °C), and steam curing regimes (90 °C) were adopted for concrete.

For the warm-water curing regime, the specimens were demolded after 24 h of casting, and then pre-conditioned under water at room temperature for one day. Finally, the samples were stored in warm water under 45 °C until testing.

For the steam curing regime, the samples were placed into a plastic tube and sealed, and then cured under a steam condition. As recommended by Zou, et al. [24]. A steam curing regime with a temperature of 90 °C and a total duration of 15 h was adopted, including a 6 h procuring period (20 °C and 95% RH), followed by a 4 h heating period at 10 °C/h up to the setting temperature and then kept for 12 h, with subsequently, 2 h cooling down period. After steam curing, the concretes were demolded and placed in a curing room ((20 ± 1) °C, RH ≥ 95%) before testing.

### 2.4. Test Methods

The slump flow and the air content of fresh mortar mixture was measured using the water column method, as recommended by Mo et al. [3].

Mechanical properties of the specimen were characterized by flexural and compressive strength following the Chinese National Standard GB/T 17671-1999 [25]. The final flexural strength was obtained by the average of three readings. The final compressive strength was determined by the average of the six broken samples from the flexural strength test.

XRD, FTIR, and SEM measurements were stopped at designated ages. For this purpose, particles with sizes of around 3 mm cut from the prism specimen were placed in a cylindrical plastic bottle with a diameter of 80 mm and a height of 60 mm. Then these bottles were filled with ethanol and sealed continuously for seven days. Finally, these samples were dried in a vacuum oven at 60 °C for 48 h for excluding residual ethanol before SEM measurement. Moreover, the dry powders (<80 μm) were produced by grinding these dried samples before XRD and FTIR measurements. 

The constituent analysis of the powder specimens was conducted by X-ray diffraction (XRD, D8, ADVANCE, Ettlingen, Germany) using a diffract meter (Cu K_α_, λ = 1.54 Å) at 40 kV and 35 mA. The diffraction patterns were obtained between 10 and 70 degrees. The chemical structures of the concrete containing ITP under different curing conditions were investigated b Fourier transform infrared spectroscopy (FTIR BRUKER TENSOR II, Ettlingen, Germany). The scanning electron microscopy (SEM, Zeiss, Gemini 300, Jena, Germany) was used to analyze the morphology and the internal microstructure of concrete containing ITP under different curing conditions selected small pieces of samples (~3 mm thick) At last, the surface of the samples was coated with Au to prevent charging effects before testing. The test condition was in a vacuum and the test voltage was 15 kV.

## 3. Results and Discussion

### 3.1. Compressive Strength

#### 3.1.1. Effect of ITP Content

**Figure 3:**
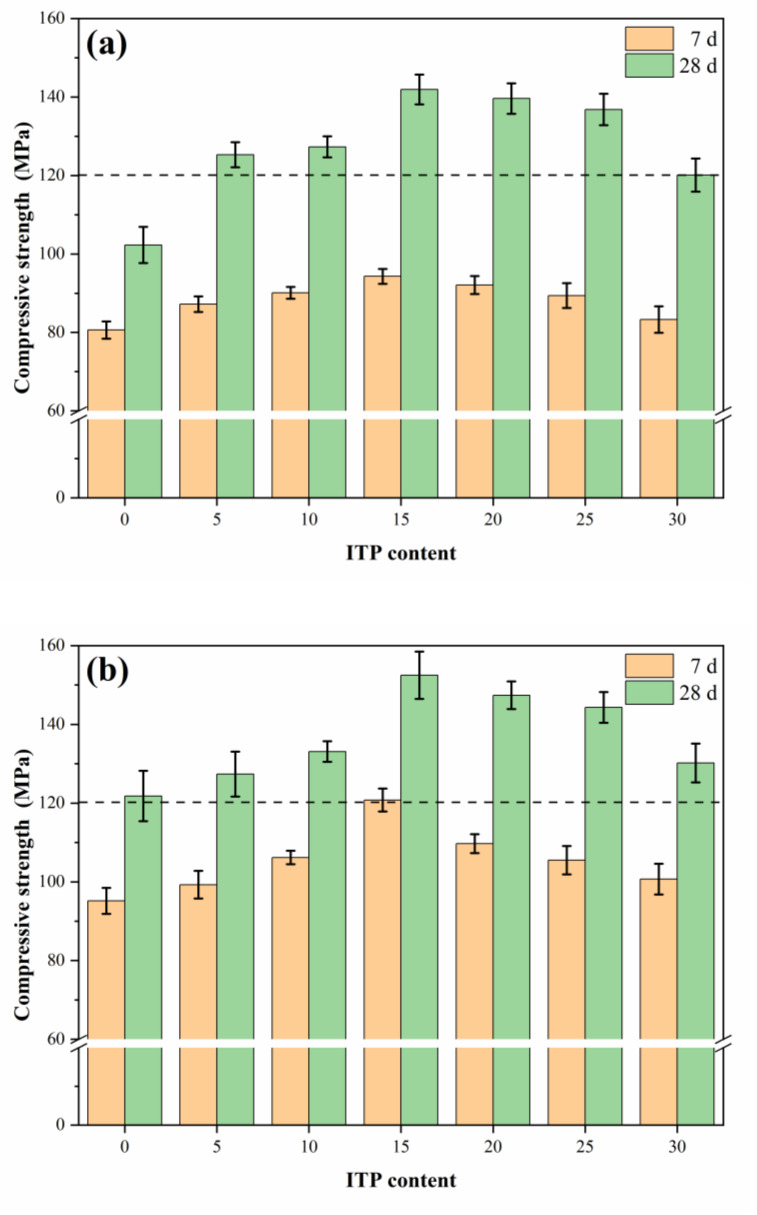

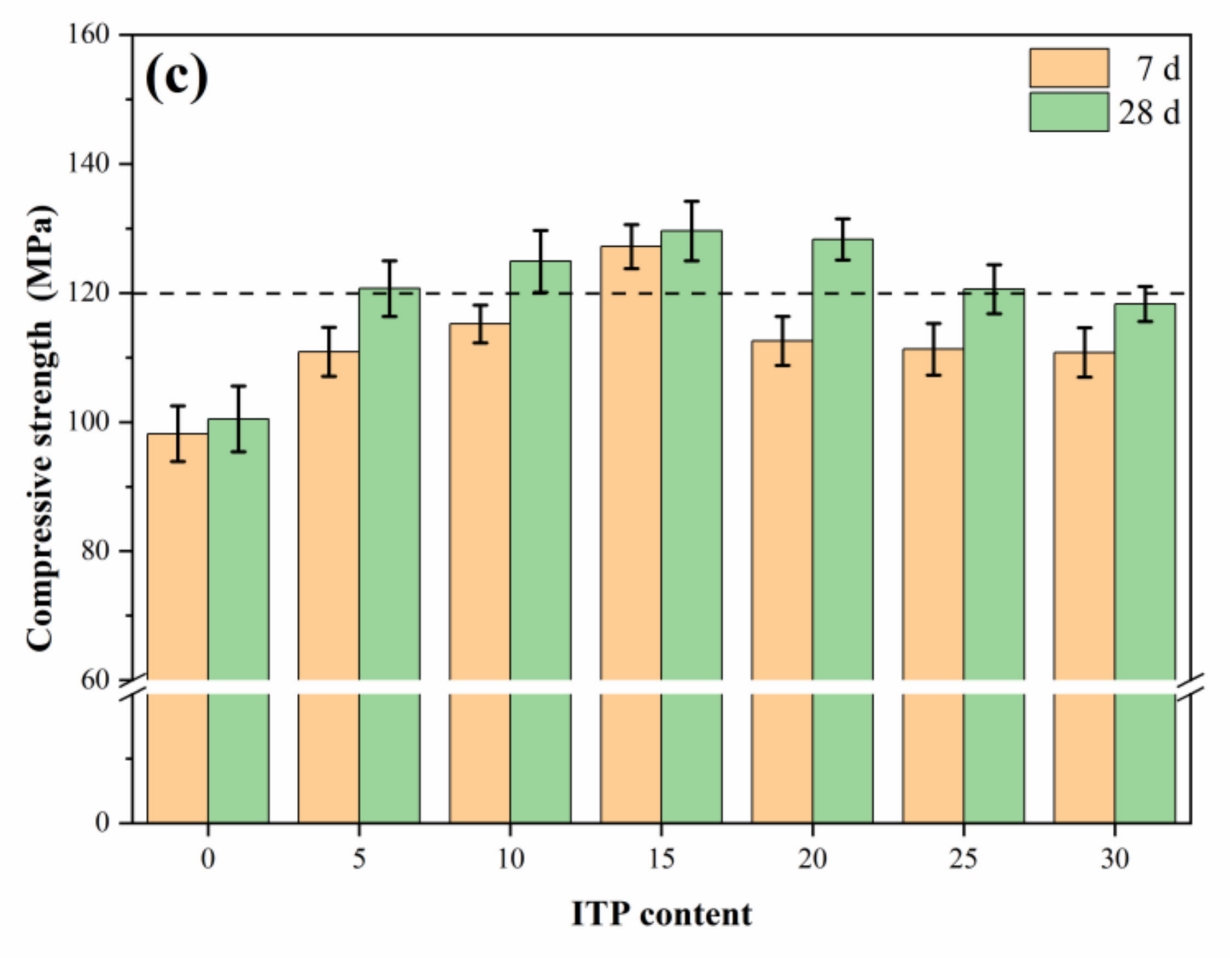
Compressive strength of samples containing ITP under (**a**) standard curing; (**b**) warm-water curing; and (**c**) steam curing.

The compressive strength of concrete improved first and then decreased with the ITP content, and reached its maximal value with 15% ITP (Figure 3). After 28 days of curing, the concretes with 15% ITP obtained 141.9 MPa (standard curing), 152.5 MPa (warm-water curing), and 129.6 MPa (steam curing), respectively. The nucleation effect and filler effect [23,26] of ITP accelerated the hydration of cement [27], formed a larger amount of hydration products (C-S-H gel and AFt) [16], and contributed to their compressive strength. Also, the small particles of ITP can fill the pores and refine the pore structure [20].

Whereas the compressive strength of the concretes with 30% ITP slightly decreased by 1.6% (7 days) and 3.1% (28 days), compared to the sample without ITP under standard curing. The ITP with high fineness (as illustrated in Section 2.1), which adsorbed a lot of free water. In consequence, increased the amount of unhydrated cement and decreased the strength. The number of hydrates generated by ITP was limited owing to its low reactivity [26]. Portland cement was substituted by ITP decreased the number of hydration products. Therefore, adding ITP could increase its strength, but there might exist an optimum ITP content (15%).

#### 3.1.2. Effects of Curing Regimes

The compressive strength of samples under standard curing all exceeded 120 MPa (except for the reference concrete without ITP at 28 days), see Figure 3a. Which indicated that the addition of ITP (within 30%) could obtain the outstanding compressive strength. Furthermore, the compressive strength of the specimens under standard curing significantly increased by 26.9% (C100ITP0), 50.5% (C85ITP15), and 44.2% (C70ITP30), compared with that of the samples at 7 days. This is expected because standard curing helps to fully hydrated cement particles.

Figure 3b shows that the warm-water curing can both significantly improve their early-age and later-age compressive strength, compared with the samples without ITP. For example, the compressive strength of the specimens with 15% ITP dramatically increased by 26.9% (7 days) and 25.2% (28 days), respectively. That is expected because of the advanced hydration of cement when the samples are immersed in warm water. Sufficient moisture and high-temperature help to hydration of cement, which resulted in a denser microstructure (will be discussed in Section 3.5).

Figure 3c shows that the steam curing regime can further enhance their early-age compressive strength, while was a limited improvement of their later-age compressive strength. This attributed to the activity of ITP was greatly stimulated under a steam curing regime [26]. As well as the hydration reaction of cement was accelerated at high temperature [28], which makes the structure denser and arrange neatly (will be discussed in Section 3.5) [29]. Moreover, the previous reports have confirmed that steam curing can significantly refine the pore structure [13]. In contrast, the compressive strength of the concretes at 28 days slightly increased to 100–130 MPa. The steam curing enhanced the cement hydration at an early age, the surface of the particles was quickly covered by cement hydrates, which hinders further hydration of cement at a later-stage [29].

Additionally, the compressive strength of the concretes with 15% ITP significantly increased by 28.1% (warm-water curing) and 34.9% (steam curing), compared with the sample C85ITP15 at 7 days under standard curing. While slightly increased by 7.5% (warm-water curing) and decreased by 8.7% (steam curing) at 28 days. This is consistent with the conclusion of Benammar et al. [30]. Additionally, a large number of large pores were formed due to the application of steam curing [31], which resulted in the concrete under steam curing was detrimental to the strength development at a later-age.

### 3.2. Flexural Strength

**Figure 4:**
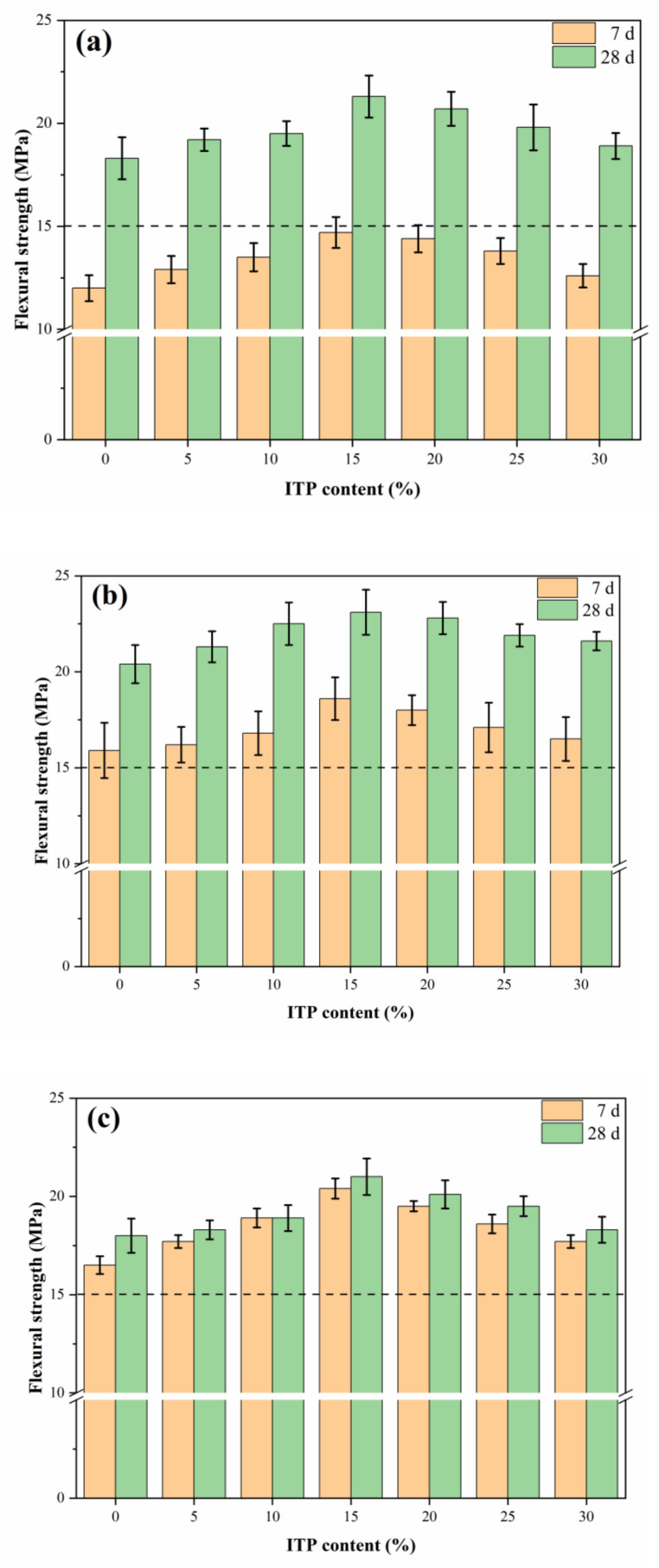
Flexural strength of samples containing ITP under (**a**) standard curing, (**b**) warm-water curing; and (**c**) steam curing.

The specimens with 15% ITP obtained the highest flexural strength (14.7 MPa for 7 days and 21.3 MPa for 28 days) under standard curing (Figure 4). Moreover, the flexural strength of the sample C70ITP30 slightly decreased by 5.0% (7 days) and 3.3% (28 days), respectively, compared with the sample C100ITP0 under standard curing. This further confirmed that there existed an optimum ITP content (~15%) for improving the strength of concrete. Similar conclusions have been illustrated by other reports [32].

Early-age flexural strength of concrete could be significantly enhanced at 7 days. For example, the concrete with 15% ITP significantly increased by 22.5% (standard curing), 55.0% (warm-water curing), and 70.0% (steam curing), compared with the specimen without ITP under standard curing (Figure 4). Interestingly, the standard curing and warm-water curing regimes significantly improve the flexural strength in the later-age, while the steam curing has a limited improvement of their later-age flexural strength. The concretes under steam curing showed lower flexural strength, compared with that of the samples under standard curing and warm-water curing regimes.

### 3.3. Hydration Products Analysis

**Figure 5:**
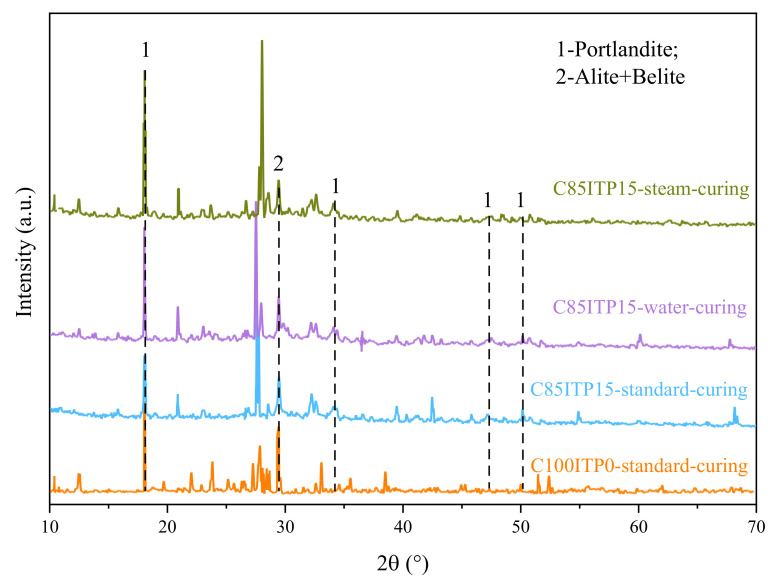
Hydration products of C100ITP0 under standard curing and C85ITP15 under different curing procedures (only the characteristic peaks of portlandite, alite, and belite are marked in the patterns).

The diffraction peaks of portlandite (CH) in sample C85ITP15 was weaker than that of the reference sample (C100ITP0) under standard curing (Figure 5). This was mainly due to replacing a portion of cement with ITP diminishes the diffraction peak of portlandite. Weaker diffraction peaks of alite (C_2_S) and belite (C_3_S) were observed in samples C85ITP15. This observation confirmed that adding ITP promoted the hydration of alite and belite and helped to the formation of more hydration products [33]. These results are following the findings of Han et al. [16]. The peaks of alite and belite in C85ITP15 under standard curing are weaker than that of samples under two other curing regimes. This result confirmed that warm-water curing and steam curing accelerate the hydration of cement, help to form hydration products and improve the early-age strength of concrete. It agrees well with the above mechanical strength test results (Figure 3 and Figure 4).

### 3.4. Chemical Structures Analysis

**Figure 6:**
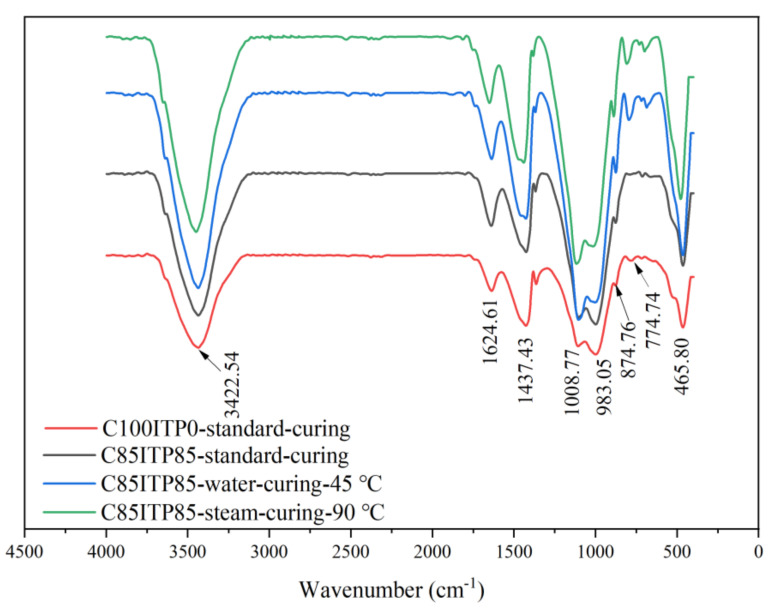
FTIR curves of C100ITP0 under standard curing and C85ITP15 under different curing procedures.

As seen in Figure 6, the absorption band around 3422.54 cm^−1^ was ascribed to O-H stretching vibration of structural water in ettringite (AFt) and calcium-silicate-hydrate (C-S-H) gel [34,35]. While the absorption peak around 1624.61 cm^−1^ was attributed to the H-O-H vibration of interlayer water [36]. Moreover, the absorption peak of O-H in C85ITP15 samples was considerably stronger than that of sample C100ITP0 at 7 days, which is in line with the XRD results (see Section 3.3). It indicates that adding ITP increased the number of hydration products (Figure 5) and enhanced the early-age strength of concrete (Figure 3 and Figure 4).

Additionally, the absorption peak at 1437.43 cm^−1^ was associated with asymmetric stretching vibration of C-O in calcite [16], the presence of calcite could be attributed to the carbonation of the cement paste during sample preparation [16]. This absorption peak in C85ITP15 under steam curing was considerably stronger than that of the sample under the two other curing regimes. The absorption band of S-O at 1008.77 cm^−1^ corresponded to Si-O stretching vibrations of Si-O tetrahedron [37], which indicates that the formation of the C-S-H gel [38]. The absorption peak at 983.05 cm^−1^ corresponded to the stretch vibration of Si-O also confirmed that the formation of C-S-H gel [16]. Notable that, the absorption peak of S-O at 1008.77 cm^−1^ in C85ITP15 was stronger than C100ITP0, which indicates that adding ITP resulted in the formation of a larger amount of C-S-H gel. Moreover, warm-water curing and steam curing regimes further increased the quantity of C-S-H gel in C85ITP15. It agrees well with the above XRD analysis (Figure 5). The absorption peak at 874.46 cm^−1^ related to the asymmetric stretching vibration of Al-OH in AFt [37]. It can be observed that the absorption peak around 874.46 cm^−1^ in C85ITP15 under steam curing was slightly stronger than that of the samples under standard curing at 7 days. This indicates that steam curing was more effective than standard curing in improving the early-age strength of concrete.

### 3.5. Microstructure Observation

**Figure 7:**
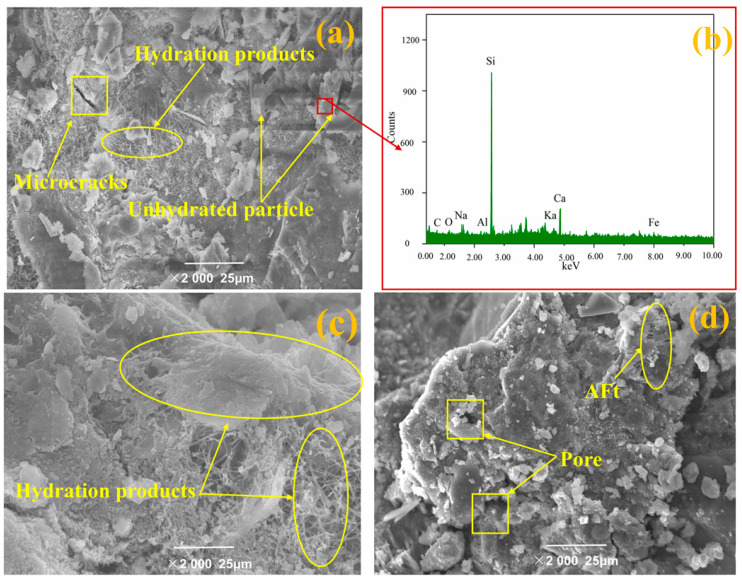
The microstructure of concrete: (**a**) the C100ITP0 sample; (**b**) XRD analysis of the unhydrated particle (red square), it indicates that the unhydrated particle was ITP; (**c**) C85ITP15 sample, and (**d**) C70ITP30 sample.

The microstructure of concrete containing different contents of ITP is presented in Figure 7, showing that there is a large number of cement hydrates and few voids. Furthermore, some unhydrated particles can be found in sample C100IP0 (Figure 7a). It can be inferred that the unhydrated particles are cement clinker through EDS analysis (Figure 7b). The sample C85ITP15 appeared a remarkably dense microstructure, there are a large number of clustered and needle-like hydration products (Figure 7c). Furthermore, the surface of the ITP particle is not smooth, and which is helpful links with the cement hydrates. Therefore, adding 15% ITP optimizes the pore size of the specimen and helps to improve their strength. This result is consistent with the above-discussed results (see Section 3.1). However, the microstructure of concrete with 30% ITP was loose and porous (Figure 7d). The fine ITP particles have a high specific surface area that can adsorb a lot of free water, increasing the amount of unhydrated cement. Moreover, the chemical reactivity of ITP was lower than that of the cement, the addition of overdosage of ITP (30%) decreased the number of hydration products. 

**Figure 8:**
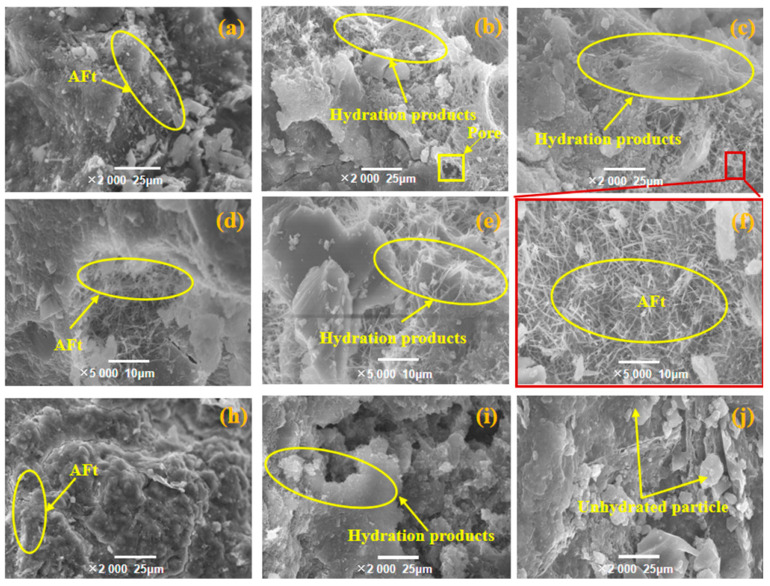
Microstructure of C85ITP15 under (**a**) standard curing; (**b**) warm-water curing and (**c**) steam curing at 7 days; (**d**) 5000 times magnification of the sample C85ITP15 under standard curing; (**e**) partially enlarged view for the C85ITP15 under warm-water curing and (**f**) partially enlarged view for the sample C85IP15 under steam-curing (red square); The sample C85ITP15 under (**h**) standard curing; (**i**) warm-water curing and (**j**) steam curing at 28 days. AFt refers to the ettringite.

The microstructure of the samples C85IP15 under warm-water curing (Figure 8b) and steam curing (Figure 8c) had more hydration products and denser structure, compared with that of the sample under standard curing at 7 days (Figure 8a). This was attributed to the reactivity of ITP, that is greatly stimulated under the steam curing regime [1]. Additionally, the hydration reaction of the cement is accelerated at elevated temperature [8], which makes the structure denser and improves the strength of concretes (see Section 3.1). Upon further amplification of the acicular hydration products that exist in C85IP15, there are a lot of cluster structures and some acicular hydration products even formed interpenetrating networks (Figure 8f). The ITP has reactivity after grinding, which has the potential to react under the stimulation of CH and gypsum [17]. Moreover, the unreacted particles form a good grade and contribute to the dense structure [18], which provides excellent strength and helps to improve the concrete strength. Thus, the concrete incorporating 15% ITP under steaming curing at 45 °C obtained the highest early-age compressive strength (Figure 3a). Whereas, there are a large number of unhydrated cement particles that could be observed in the sample C85ITP15 at 28 days (Figure 8h). Thus, the steam curing at 90 °C is detrimental to the microstructure and the later-age strength formation of the concrete, compared with the standard curing (Figure 8i) and warm-water curing (Figure 8j).

## 4. Conclusions

This paper has designed seven UHPC formulations incorporating different contents of ITP and investigated the effects of curing conditions on mechanical and microstructural properties of UHPC. The following conclusions can be made:

1. Compressive and flexural strength of UHPC improved first and then decreased with the ITP content and reached its maximal value with about 15% ITP. Compressive strength of concrete at 28 days exceeded 120 MPa (except for the samples C100ITP0-standard-curing, C100ITP0-steam-curing, and C70ITP30-steam-curing).
2. The warm-water curing and steam curing regimes were more effective than standard curing in improving the early-age mechanical properties of concrete. The following ranking of the curing regimes through the test analysis is recommended: steam curing > warm-water curing > standard curing. While the warm-water curing has the best effect on the later-age strength improvement of concrete, followed by standard curing, and then steam curing regime.
3. The addition of overdosage of ITP (30%) increased the amount of unhydrated cement and decreased the number of hydration products. The steam curing at 90 °C has detrimental to the microstructure of concrete and tended to a higher porosity.
4. The filler effect of ITP, the reactivity of ITP stimulated under the steam curing regime, and the hydration rate of cement under high temperature are key parameters affecting the microstructure evolution and mechanical properties of concrete.

## Figures and Tables

**Table 1:** Physical properties of cement.

Flexural STRENGTH (MPa)		Compressive Strength (MPa)		Specific Surface Area (m^2^/kg)	Density (g/cm^3^)
3 d	28 d	3 d	28 d		
4.2	8.3	23.7	46.7	350	3.10

**Table 2:** Chemical composition of cementitious materials (wt.%).

Composition	CaO	SiO_2_	Al_2_O_3_	Fe_2_O_3_	MgO	SO_3_	Loss on Ignition (%)
Cement	57.58	20.35	6.12	4.23	2.59	2.19	2.58
Silica fume	0.41	94.02	0.27	0.11	0.34	0.11	2.86
Iron tailing powder	12.12	51.85	11.24	9.34	4.86	0.41	2.35

**Table 3:** Mix proportion of the reference sample (kg/m^3^).

Cement	Silica Fume	Iron Tailing Powder	Quartz Sand	Superplasticizer
672	168	0	1330	21

**Table 4:** Mix proportions for UHPC.

Samples ID	w/b	Portland Cement Was Substituted by ITP (wt.%)		Flow Spread (mm)	Air Content (%)
		Cement	ITP		
C100ITP0	0.25	100	0	285	7.82
C95ITP5		95	5	275	7.75
C90ITP10		90	10	265	7.63
C85ITP15		85	15	260	7.55
C80ITP20		80	20	255	7.39
C75ITP25		75	25	245	7.32
C70ITP30		70	30	245	7.26

## Data Availability

Data sharing is not applicable to this article.
